# Future of Probiotics and Prebiotics and the Implications for Early Career Researchers

**DOI:** 10.3389/fmicb.2020.01400

**Published:** 2020-06-24

**Authors:** Irina Spacova, Hemraj B. Dodiya, Anna-Ursula Happel, Conall Strain, Dieter Vandenheuvel, Xuedan Wang, Gregor Reid

**Affiliations:** ^1^Laboratory of Applied Microbiology and Biotechnology, Department of Bioscience Engineering, University of Antwerp, Antwerp, Belgium; ^2^Department of Neurobiology, The University of Chicago, Chicago, IL, United States; ^3^Division of Immunology, Department of Pathology, Institute of Infectious Disease & Molecular Medicine, University of Cape Town, Cape Town, South Africa; ^4^APC Microbiome Ireland, University College Cork, Cork, Ireland; ^5^Teagasc Food Research Centre, Fermoy, Ireland; ^6^Institute for Biomedical Sciences, Shinshu University, Nagano, Japan; ^7^Department of Zoology, University of Oxford, Oxford, United Kingdom; ^8^Department of Biochemistry, University of Oxford, Oxford, United Kingdom; ^9^Centre for Human Microbiome and Probiotic Research, Lawson Health Research Institute, London, ON, Canada; ^10^Department of Microbiology and Immunology, The University of Western Ontario, London, ON, Canada; ^11^Department of Surgery, The University of Western Ontario, London, ON, Canada

**Keywords:** probiotics, prebiotics, review, young scientist, ISAPP, SFA

## Abstract

The opportunities in the fields of probiotics and prebiotics to a great degree stem from what we can learn about how they influence the microbiota and interact with the host. We discuss recent insights, cutting-edge technologies and controversial results from the perspective of early career researchers innovating in these areas. This perspective emerged from the 2019 meeting of the International Scientific Association for Probiotics and Prebiotics - Student and Fellows Association (ISAPP-SFA). Probiotic and prebiotic research is being driven by genetic characterization and modification of strains, state-of-the-art *in vitro*, *in vivo*, and *in silico* techniques designed to uncover the effects of probiotics and prebiotics on their targets, and metabolomic tools to identify key molecules that mediate benefits on the host. These research tools offer unprecedented insights into the functionality of probiotics and prebiotics in the host ecosystem. Young scientists need to acquire these diverse toolsets, or form inter-connected teams to perform comprehensive experiments and systematic analysis of data. This will be critical to identify microbial structure and co-dependencies at body sites and determine how administered probiotic strains and prebiotic substances influence the host. This and other strategies proposed in this review will pave the way for translating the health benefits observed during research into real-life outcomes. Probiotic strains and prebiotic products can contribute greatly to the amelioration of global issues threatening society. The intent of this article is to provide an early career researcher’s perspective on where the biggest opportunities lie to advance science and impact human health.

## Introduction

Trillions of microbes inhabit the human body, collectively forming the human microbiota. These microbes create complex, organ-specific and adaptive ecosystems, which continually impact the host’s physiology. The microbiota and its overall genetic material (the microbiome) consist of bacteria (bacteriome), archaea (archeome), fungi (mycobiome), viruses (virome), and parasites (parasitome). Together they play a pivotal part in human and animal physiology through influencing digestion, immune development, vitamin production, and likely behavior and mental wellbeing (Belkaid and Hand, 2014; Biesalski, 2016; Borre et al., 2014; Leung et al., 2018). Beneficial effects of certain microorganisms are reflected in the concept of probiotics, defined as “live microorganisms that, when administered in adequate amounts, confer a health benefit on the host” (Hill et al., 2014). Also, certain microbial substrates, called prebiotics, can be selectively utilized by host microorganisms, thereby conferring a health benefit (Gibson et al., 2017). Thus, by supplementing with appropriate probiotics and/or prebiotics, it is possible to provide added benefits to human health.

**FIGURE 1:**
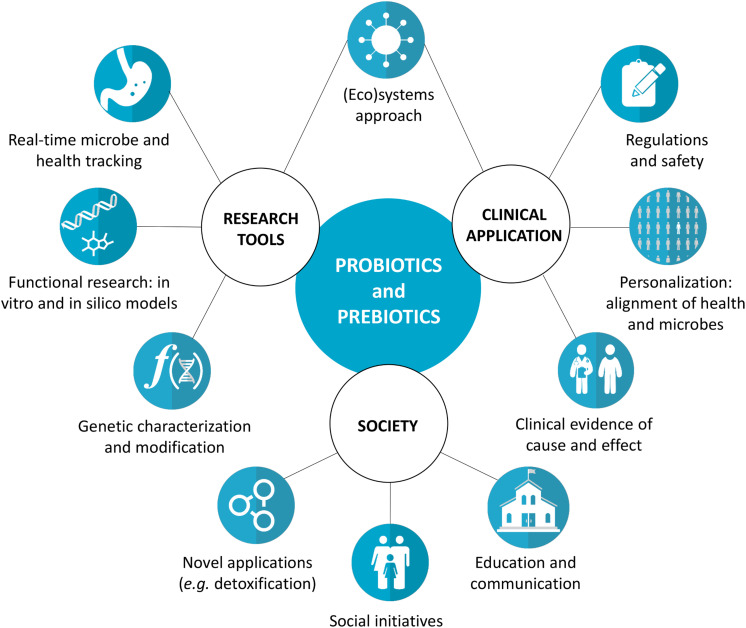
Key concepts for the development of the probiotics and prebiotics fields. We propose a systems approach linking research tools and clinical application to translate the potential health benefits observed during research into real-life outcomes. For example, not only the probiotics and prebiotics themselves should be considered, but also the real-time interactions with the host, microbial functionality, individual factors, and the regulation and safety aspects. Furthermore, various environmental and social needs can and should be addressed by probiotics and prebiotics.

The fields of probiotics, prebiotics, and related microbiome research have seen remarkable advances in the last decades (Sanders et al., 2019). Novel tools offer alternatives to traditional *in vitro* and *in vivo* models (Table 1), allowing more efficient generation of convincing data on the probiotic and prebiotic mode of action, their effect on the microbiome, and the resulting clinical health outcomes. Numerous clinical trials provide evidence of strain-specific probiotic and prebiotic benefits for a range of health conditions, including diarrhea (Guandalini, 2011) vaginal dysbiosis (Reid et al., 2003), respiratory infections (Hatakka et al., 2001), bowel function (Vandeputte et al., 2017) body weight (Nicolucci et al., 2017) and bone mineralization (Abrams et al., 2005). These findings have sparked an interest from the press and general public, emphasizing the need for correct and accessible scientific communication around these topics. Taken together, this calls for a discussion on the research possibilities and implementation of probiotics and prebiotics and their interaction with the host microbiome (Figure 1).

**TABLE 1:** Promising in *vitro, in vivo*, and *in silico* techniques for probiotics and prebiotics research.

Technique	Use	Advantages	Limitations	References
**Microbiome and *in silico* methods and models**				
Full shotgun metagenomics sequencing	To sequence the genomes of untargeted cells in a community to elucidate community composition and function	Untargeted, allows simultaneous detection of bacterial, fungal and viral sequences, greater taxonomy resolution and functional profiling	Expensive, requires more extensive data analysis due to host DNA interference	Laudadio et al. (2019)
Strain-specific quantitative polymerase chain reaction (qPCR)	To quantify target DNA sequences for specific probiotic strains	Faster high-throughput detection and quantification of target DNA sequences, high sensitivity enables quantification of microorganisms with low abundance on strain level within an environmental sample	Design of primers that specifically target strains of interest despite the presence of closely related strains is not trivial, requires adequate validation, results need to be correlated with phenotypic and biochemical tests	Treven (2015), Maldonado-Gómez et al. (2016)
KatharoSeq	High-Throughput Microbiome Analysis of Low-Biomass Samples	Able to differentiate a true positive signal in samples with as few as 50 bacterial cells, high-throughput, single tube DNA extractions, automated, incorporates positive and negative controls, combines laboratory and bioinformatic methods	Careful selection of positive controls necessary	Minich et al. (2018)
RIDE checklist	Minimum standards checklist for low microbial biomass microbiome studies	Improves the validity of low microbial biomass research by reporting methodology, including controls, determining level of contamination and exploring impacts of contamination in downstream analysis	Sample collection recommendations difficult to implement in some clinical settings	Eisenhofer et al. (2019)
**Metabolite/protein detection methods**				
^1^H-NMR-spectroscopy	Detection of metabolites in biological samples	Not destructive, minimal sample preparation, broad	Less sensitive than mass spectrometer-based methods, quantification of metabolites challenging	Beckonert et al. (2007)
Targeted Tandem Mass Spectrometry (TQ, QTrap)	Targeted analysis of metabolites, hypothesis-driven research	High sensitivity and specificity, absolute quantification	Limited spectrum of metabolites, risk of false positives	Gowda and Djukovic (2014), Schrimpe-Rutledge et al. (2016)
Untargeted Tandem Mass Spectrometry (Q-TOF, LTQ-Orbitrap)	Global profiling of metabolites, hypothesis-generating research	Comprehensive analysis, can detect unknown metabolites	Relative quantification, libraries for annotating incomplete, risk of false negatives.	Gowda and Djukovic (2014), Schrimpe-Rutledge et al. (2016)
Proteomics	Detection of expressed proteins	Untargeted, direct method, high sensitivity, allows identification of human and bacterial proteins	Low throughput, time consuming, requires known peptide annotation, likelihood of 100% amino acid sequence identity between proteins produced by different species is low, same protein might be expressed by various organisms	Verberkmoes et al. (2009)
**Genetic manipulation**				
CRISPR-Cas9	Targeted genetic manipulation	Efficient and specific, limited off-target mutation, no need for a permanent antibiotic marker	Often demands subsequent transformations, limited to genetic sites with a PAM motif present	van Pijkeren and Britton (2014), van Pijkeren and Barrangou (2017)
Food-grade cloning vectors	Genetic manipulation of food-grade probiotics for safe use in humans and animals	To create genetically modified probiotics that meet the non-toxic and safe for consumption criteria (e.g., isogenic probiotic mutants for mechanistic studies in humans)	Limited functional marker genes	Landete (2017), van Pijkeren and Barrangou (2017)
Genetic modification of probiotics to produce therapeutic molecules	Next-generation probiotics as delivery vehicles for bioactive compounds or antigens	Combination of beneficial probiotic action with targeted delivery of therapeutic molecules aimed against specific diseases	Approval for general use under current regulation is challenging, need for extensive safety testing and strict biocontainment strategies	Lagenaur et al. (2011), Bron and Kleerebezem (2018)
***In vitro/ex vivo* techniques, tissue and organ models**				
RNAseq	Measures gene transcription in bacterial communities and host, provides information about gene expression under different ecological conditions	Allows to determine transcriptional responses, not limited to genomic sequences, quantifiable	Dependent on successful cDNA synthesis, challenging for short-lived transcripts	Mortavazi et al. (2008)
Explants and organotypic tissue models	Assessment of safety, mechanisms of action and potential efficacy of probiotic candidate	Three-dimensional tissue structures, differentiated cell composition, reflective of human physiology	Limited culture time and thus limited potential for long-term studies, not suitable for predicting systematic effects	Lagenaur et al. (2011), Ñahui Palomino et al. (2017)
Organoids	Assessing probiotic efficacy and mechanisms of action at organ-level biological read-outs	High reproducibility, recapitulation of 3D physiological structures	Variability in cell types/heterogeneity, less appropriate for studying effects on stratified tissues	Han et al. (2019)
Microfluidic organ-on-a-chip models	Kinetic assessment of prebiotic, probiotic and microbiota effects on host cells	Reflect the physiological complexity of dynamic niches, allow kinetic read-outs	Technologically challenging	Bein et al. (2018), Greenhalgh et al. (2019)
Mini bioreactors	Study metabolites and capture community changes	Traditional fermentation model, high throughput, reduced volume	No precise pH control in place, no distinct compartments of the colon, no interactions with host cells	Auchtung et al. (2016)
***In vivo* approaches**				
Humanized animal models	Evaluating microbe-host and prebiotic-host interactions	Increased translational value compared to traditional animal models, more accurate modeling of specific human-like host responses, possibility to colonize animals with defined probiotic strains or human microbiota	Not representative from a host-specific evolutionary perspective, technical challenges (e.g., graft rejections of human microbiota or immune cells), ethical concerns	Martin et al. (2008)
Osmotic pill	Real-time *in vivo* microbiome sampling along the gastrointestinal tract	Ingestible, biocompatible and battery-less with an osmotic sampler and microfluidic channels, allows real-time *in vivo* sampling along gut lumen	Collection time is variable and influenced by individual peristaltic movement	Nejad et al. (2019)
Randomized controlled trials	Assessment of prebiotic and probiotic intervention outcomes under controlled conditions	Gold standard to assess intervention outcomes in humans	Expensive, time-consuming, randomization might prevent patient stratification based on relevant personal parameters, outcomes might be different in different patient populations	Anukam et al. (2006), Sanchez et al. (2014), Panigrahi et al. (2017)

## Developments in Probiotics and Prebiotics Research

### Considerations for Probiotics Research

#### Genetic Characterization and Modification of Probiotics

Bioinformatics and *in silico* approaches contribute to a more detailed understanding of beneficial microorganisms, thereby allowing their targeted usage and safety assessment. As whole genome sequencing (WGS) is available at a reasonable cost, we advise that qualitative WGS and rigorous annotation should become the standard practice prior to marketing new probiotic strains. Newly sequenced genomes should be deposited and made publicly available via standard central databases (e.g., GenBank^1^, DDBJ^2^, ENA^3^). A rigorous sequence quality control and annotation should be carried out (Smits, 2019) identifying the mobile and other genetic elements (e.g., CRISPR arrays) and predicting their functional properties, thereby estimating the safety of probiotic candidates regarding virulence factors and possible antibiotic resistance gene transfer. The European Food Safety Authority (EFSA) and recent publications recommend WGS to improve the monitoring of foodborne antimicrobial resistance (Collineau et al., 2019; Efsa et al., 2019) and workflows to assess risk-related gene traits based on WGS are available (Salvetti et al., 2016). WGS is also useful to assess genetic instability and ensure the retention of regions linked to the strain’s health benefits, as demonstrated for the *Lacticaseibacillus rhamnosus* GG variants with and without *spaCBA* pili genes (Sybesma et al., 2013).

Genetic manipulation is an important tool to study probiotic mechanisms of action [e.g., by using isogenic mutant strains (Lebeer et al., 2011)] and to potentially create improved strains (Lebeer et al., 2018; Petrova et al., 2018). However, a lack of sufficient genetic tools available for some probiotic species, especially food-grade systems for bifidobacteria, and a legal framework for the use of genetically manipulated/enhanced organisms limits the research progress (Allain et al., 2015; Asto et al., 2019). In addition to ethical and legal questions, it is not yet common practice to send genetic constructs to biological repositories. The considerable effort it takes to make these constructs and regulations within the research institutions are probably the main reasons to keep this genetic material in-house. We therefore advocate for a better sharing of genetic constructs by providing the material to the scientific community through existing repositories (e.g., AddGene, European Nucleotide Archive). This can save a substantial investment of time and research funds and will serve to enlarge the genetic tools box for probiotics. In particular, the development of safe vectors (e.g., food-grade vectors) is a necessary step in genetically tweaking probiotic strains for industrial and pharmaceutical applications (Landete, 2017), including specialized probiotics designed to deliver bioactive compounds to more effectively target specific diseases (Table 1). Both fundamental and applied probiotics research would benefit from a vast investment in genetic elements, and a better understanding of their mechanism of action and specificity (Allain et al., 2015). Genetic and bioinformatic training and experience in these interdisciplinary areas will thus be key for researchers making progress in the probiotics field.

#### *In vitro* Models in Probiotics Research

Animal models are not strictly necessary for preclinical assessment of probiotics. While humanized animal models can be implemented (Table 1), it is highly challenging to develop ones that simulate microbe-host interaction in humans for niches demonstrating unique physiological features such as the lactobacilli-dominated vaginal niche characterized by a low pH (Miller et al., 2016). Human-based *in vitro* and *ex vivo* models followed by small studies with healthy volunteers and larger clinical intervention studies are invariably required to draw more precise and relevant conclusions on probiotic safety, action, and health benefits.

Recent cutting-edge *in vitro* and *ex vivo* approaches based on human cells and tissues pave the way beyond *in vitro* cell lines and animal models (Table 1). Reproducible human organoids have been used to recapitulate irritable bowel syndrome manifestations and the restorative effects of *L. rhamnosus* GG (Han et al., 2019). Sophisticated organs-on-chips combine advances in human cell culturing with microelectronics and microfluidics to discover the anti-cancer potential of probiotic and synbiotic formulations, however, they are limited due to lack of exposure to host defenses (Bein et al., 2018; Greenhalgh et al., 2019). Cervico-vaginal tissue explants and organotypic tissue models circumvent this by combining human epithelial and immune cells and have previously allowed to identify anti-HIV-1 effects of wild type and genetically modified lactobacilli (Lagenaur et al., 2011; Ñahui Palomino et al., 2017).

It is notable that on September 10, 2019, the United States Environmental Protection Agency released a memorandum stating that studies on mammals will be eliminated by 2035. This has implications for early career scientists developing their future line of research, opening opportunities for implementation of alternative *in vitro* models.

### Considerations for Prebiotics Research

#### *In vitro* Methods to Study Prebiotics and Their Targets

Early prebiotic research was focused on the ability of compounds to stimulate bifidobacterial growth in the gut. Now, research has expanded to prebiotics targeting other species in the gut, but also in the vagina, lung, and skin (Gibson et al., 2017; Collins et al., 2018). This offers opportunities for prebiotics beyond dietary non-digestible carbohydrates targeting colonic microbiota. Studies are warranted to identify novel prebiotics, especially by experts in chemistry and food science, and engineers designing delivery systems. In addition, synbiotics can exert a synergistic effect on host health with promising clinical effects against neonatal sepsis (Panigrahi et al., 2017) and insulin resistance (Eslamparast et al., 2014).

A diverse array of *in vitro*, *in vivo*, and *in silico*-based techniques has been applied to understand the mechanisms of action and efficacy of prebiotics (Table 1). The traditional method has been a bottom-up approach, where candidate prebiotics are screened by *in vitro* techniques, such as single strain culturing and chemostat fermentation of stool samples, before employing them in animal models and human intervention studies.

A novel top-down systems biology-based approach has recently been developed through *in silico* methods, which involve computer modeling and analysis of biological processes and interactions. For this, existing metagenomics and genomic data from human studies are mined to identify potential novel beneficial species able to prosper with the compound being tested. The prebiotics for these species are then selected by metabolic models based on microbial genomic data (Shoaie et al., 2015; Tramontano et al., 2018). Attempts have been made to create metabolic models of microbial inhabitants of the gut, to unravel the complex cross-feeding that occurs within the gut microbiota (Shoaie et al., 2013). We argue that *in silico* techniques are useful complements to classical techniques, and their implementation has the potential to improve the design of human studies.

#### Importance of Metabolomics for Prebiotics

The recent expansion of the prebiotic concept to include other types of compounds in addition to non-digestible carbohydrates, such as polyphenols and certain fatty acids (e.g., polyunsaturated fatty acids), has resulted in greater emphasis on untargeted metabolome analysis (Bindels et al., 2015). Microbial biotransformation of dietary phytochemicals (e.g., polyphenols) leads to a diverse array of metabolites, more bioavailable than the parent compound. These could enhance health effects (Duenas et al., 2015). Evidence suggesting bacteria contribute to approximately 70% of fecal and 15% of serum metabolites highlights the importance of understanding metabolite origin (Visconti et al., 2019). An *in silico* approach including annotating metabolite data to host, diet or bacterial derived origins would contribute to this understanding (Shaffer et al., 2019). Proving the origin of a metabolite can be achieved *in vitro* and *in vivo* by a dual isotope/radio labeling of novel prebiotics to build databases for annotating and mapping metabolic networks through elucidating the pharmacokinetics of the prebiotics and the mechanism and site of action of the metabolites (Birkemeyer et al., 2005).

For those pursuing prebiotic research, this multi-dimensional approach should uncover how these compounds function within the diverse gut microbiome or at other sites.

## Discussion on Untangling the *In Vivo* Effects of Probiotics and Prebiotics

### Translating Effects of Probiotics and Prebiotics Into Real-Life Outcomes

Two recent publications questioned the *in vivo* benefits and safety of probiotics (Rao et al., 2018; Suez et al., 2018), but failed to provide sufficient, substantiated evidence on actual harm or lack of efficacy. Sweeping generalizations on probiotics as a whole should be avoided by properly documenting the strains used, and emphasizing their strain-specificity and mechanisms of action (Lebeer et al., 2018; Sanders et al., 2019). Probiotic strains with a safe history of use should be tested in small pilot trials in humans with detailed sampling and safety assessment, especially in high risk populations and regarding long-term effects (Langille et al., 2013; Sanders et al., 2019). When strains pose a safety concern, postbiotics, or “bioactive compounds produced by food-grade micro-organisms during a fermentation process,” might be an efficient alternative (Wegh et al., 2019). Subsequently, large well-designed and properly controlled trials in the target host are needed to obtain evidence of benefit, optimal dose, and intended clinical uses (Sanders et al., 2019).

Understanding the influence of interpersonal differences on clinical outcomes would greatly contribute to the efficacy of probiotic and prebiotic interventions (Mullish et al., 2020; Rodriguez et al., 2020). However, this is not a trivial task, and it is often not yet clear why a strain or compound is more effective in some individuals than others (Sanchez et al., 2014). Future research should focus on stratification of clinical trials based on individual characteristics of the participants, including sex, ethnicity, diet, and the functional characteristics of their microbiome. Already, the need for microbiome research to emphasize function rather than microbiota composition has been raised (Reid et al., 2019). This requires metabolomic tools and small molecule identification aligned with clinical evidence of cause and effect.

We advocate for adopting a systems approach that integrates readouts of functional microbial and health data in a range of samples (including stool, urine, blood, mucosa and saliva) from clinical trials. The readouts should focus on functional interactions with the host and how they are influenced by additional factors (e.g., drugs, pollutants, nutrients). This can be reasonably well achieved with a combination of *in silico, in vitro*, and *in vivo* approaches (Table 1). Precise metabolic modeling may predict which probiotic or prebiotic would be most likely to help an individual realize an effective personalized nutrition strategy for treating disease and promoting health (Tebani and Bekri, 2019). Response profiles to probiotic interventions can and should be evaluated in humans, even if it requires complicated tools like assessing the transcriptome. Such studies reveal that different probiotic *Lactobacillus/Lacticaseibacillus* strains induce different gene-regulatory pathways in the small intestinal mucosa of healthy volunteers (van Baarlen et al., 2011).

Approaches like this provide avenues for understanding the individual-specific effects of different probiotic strains and facilitate their rationally designed clinical application. An exciting future goal would be to develop personalized probiotics and prebiotics. However, given there is no single healthy microbiome (Lloyd-Price et al., 2016), the personalized concept may not require individual remedies, but rather sufficient options to manipulate functions shared by many.

### Probiotics, Prebiotics and the Microbiome: An Ecological Perspective

When exploring probiotic and prebiotic effects at distant sites, it is critical to consider the complex microbial ecosystems of those niches. The administered probiotic should ideally promote homeostasis. Longitudinal and body niche-specific microbiome sampling (Gajer et al., 2012) is important to assess the dynamic changes resulting from probiotic and prebiotic interventions. This is helped by non-invasive devices, such as an osmotic pill for real-time *in vivo* microbiome sampling of difficult to access niches in the gut (Nejad et al., 2019) (Table 1). This pill is ingestible, biocompatible and does not require a battery. It has an integrated osmotic sampler and microfluidic channels, allowing for real-time sampling of the gut lumen and its microbiome without the need for colonoscopy. This will help us understand the microbiome’s response to probiotic or prebiotic administration and pave the way for lab-on-a-pill devices.

The manner in which probiotic strains adjust to the conditions of the target site are largely unknown. Global gene expression profiling and studying isogenic mutants showed that exopolysaccharide production affects lactobacilli survival in the human (Marco et al., 2010) and murine (Lebeer et al., 2011) intestine. Interplay with environmental factors (e.g., medication, pollutants, nutrients) must also be investigated to truly appreciate factors that can influence probiotic strain activity and health of the host. Ultimately, an integrative approach would not simply establish dose-response relationships to treatment, but rather attempt to align what enters our system, how it is processed, and what microbes can be delivered to improve the net effect. It is thus inevitable that systematic approaches should be implemented to elucidate how an administered probiotic or prebiotic interacts with the host at various levels (e.g., the immune stimulator/modulator effect, microbiome, medication interactions).

The ecologic perspective will not be complete without identifying how changes in the relative abundance of one species impacts the relative abundances of others. Interdependency of sequencing data overlooks the real population dynamics. An ecosystem includes different interactions: co-operation, competition, exploitation, mutualism, co-dependency, commensalism, and amensalism, and these interactions can play a role in microbiome stability (Coyte et al., 2015). Importantly, the microbiome datasets obtained with high throughput sequencing are compositional, as they consist of proportions with a constant sum, therefore compositionally appropriate tools should be applied for their analysis (Gloor et al., 2017). To assess these complex interactions, evaluation of relative microbial community composition (e.g., by 16S rRNA gene or shotgun DNA sequencing) should be combined with species-specific qPCR for abundance, whole-transcriptome shotgun sequencing and proteomics to determine function (Quince et al., 2017) (Table 1). These approaches will allow to identify functional changes, adaptability and ecological interactions of probiotics and prebiotics with indigenous host microorganism communities. Large datasets generated using these multi “omics” could facilitate the hypothesis-driven studies at different levels of evidence.

### Impact Beyond the Bench

Probiotics, and to a lesser extent prebiotics, are now widely available to people in developed countries. Nevertheless, continual education is needed as too often the media or companies misrepresent what these are and what they can do (Reid et al., 2019). On a global front, people in developed and especially developing countries could benefit from probiotic products due to effects against infectious diseases, but the lack of affordable and well-documented strains is a hindrance (Kort et al., 2015). Various probiotic initiatives to better influence developing countries, including Westernheadseast.ca, Yoba4Life.org, and Yogurito (Argentina), have proven that populations in low-income regions derive benefits beyond probiotic-mediated health, as seen by facilitating economic development and fighting malnutrition with local resources (Reid et al., 2018, 2020). Every country should take action to implement programs allowing the poorest of their society access to fermented foods and probiotics that reduce the risk of key diseases: diabetes, malnutrition, infections.

We could also harness the power of beneficial microbes to ameliorate the impact of worldwide problems. A study showed that the probiotic *Lacticaseibacillus rhamnosus* GR-1 can sequester the heavy metals lead and cadmium, effectively reducing their translocation across the intestinal epithelium *in vitro* (Daisley et al., 2019). Thus, administration of certain probiotic strains may offer a simple and effective option to reduce the amount of heavy metals absorbed from foods in contaminated regions of the world. Novel marine probiotics can also help prevent ecological damage, for example by increasing coral resistance to bleaching (Rosado et al., 2019). When such applications become feasible, many opportunities will arise for early career researchers in the field of probiotics and prebiotics.

## Concluding Remarks and Future Perspectives

Current technological and methodological developments offer exciting possibilities for probiotics and prebiotics research and applications. New tools allowing real-time studies in humans and following a microbe as it integrates into an existing microbiota, as well as systems that can quantify levels of health, will drive this field forward. Read-outs on what microbes are present, their interaction with the host and the influence of environmental factors (e.g., drugs, nutrients) will become standard when going for a physical examination in the future.

Novel sampling systems will elucidate how an applied probiotic or prebiotic interacts with the host at various levels, including the immune system, metabolism and all components of the microbiome. Ultimately, an integrative approach will support a form of personalized medicine to establish dose-response relationships for treatment, but moreso attempt to align what enters our system, how it is processed, and which probiotics or prebiotics deliver the best desired effects. Mechanistic insights into effector molecules will pave the way for emerging concepts, such as postbiotics.

As early career scientists, we want to be part of a society that uses beneficial microbes to help solve global problems, such as reducing the risk and impact of disease (including viruses and pandemics) and removing drugs and toxins from our food and environment. These will be exciting times with many career paths open for probiotics and prebiotics research in the sciences and applied to many other disciplines.

## Author Contributions

All authors listed have made a substantial, direct and intellectual contribution to the work, and approved it for publication.

## Conflict of Interest

GR acknowledged providing advice to several companies that sell probiotic products. The remaining authors declare that the research was conducted in the absence of any commercial or financial relationships that could be construed as a potential conflict of interest.

## Footnotes

1 www.ncbi.nlm.nih.gov/genbank

2 www.ddbj.nig.ac.jp

3 www.ebi.ac.uk/ena
